# A comparison between hearing and tone burst electrophysiological thresholds

**DOI:** 10.1016/S1808-8694(15)30103-8

**Published:** 2015-10-19

**Authors:** Fernanda Rodrigues Pinto, Carla Gentile Matas

**Affiliations:** a Speech therapist, scholarship for technical training, Research Support Foundation, State of Sao Paulo - FAPESP.; b Assistant professor of the speech therapy course, Physical Therapy, Voice Therapy and Occupational Therapy Department, Sao Paulo University Medical School. Speech therapy course, Physical Therapy, Voice Therapy and Occupational Therapy Department, Sao Paulo University Medical School - FMUSP.

**Keywords:** evoked potentials, auditory, evoked potentials, auditory

## Abstract

Studies have reported compatibility between hearing and electrophysiological thresholds in the auditory brainstem response (ABR) with tone burst stimuli.

**Aims:**

to verify waves I, III, V and their latency times for tone bursts at 500, 1000, 2000 and 4000 Hz and at 80 dB HL, and to compare tone burst electrophysiological thresholds with those obtained from audiological and psychoacoustic evaluations.

**Methods:**

audiological, psychoacoustic and electrophysiological evaluations of 40 male and female normal hearing individuals aged between 18 and 40 years were undertaken.

**Results:**

only wave V was visualized at 80 dB HL and its latency values decreased with increased frequencies in both genders. At 1000, 2000 and 4000 Hz male subjects presented higher electrophysiological thresholds values than females at all frequencies. At 500, 1000 and 2000 Hz, electrophysiological, hearing, and psychoacoustic thresholds were statistically different in both genders.

**Conclusion:**

although ABR with tone burst stimulus is clinically applicable, further research is needed to standardize test techniques and results.

## INTRODUCTION

The central nervous system generates bioelectric activity while performing its tasks, which may be read by electrodes on the scalp. These low-amplitude potentials (100 millivolts) may be amplified, recorded and measured by techniques such as electroencephalography.^1^

Auditory evoked potentials may be subdivided into early, middle and late components.^2^ The brainstem auditory evoked response (BAER) is an early potential occurring 0 to 10 milliseconds (ms) after the presentation of an acoustic stimulus. Its presence or absence makes it possible to assess the integrity of brainstem auditory pathways.^3^ Responses are generated to acoustic stimuli presented through supra-aural earphones (TDH 39 model). Surface electrodes placed on the scalp and mastoids or the earlobes record the electrical activity originating in auditory pathways from the auditory nerve to the midbrain.

These responses consist of a series of seven waves generated by one or more structures along the auditory pathways that have the following generating sites: wave I: the portion of the auditory nerve distal to the brainstem; wave II: the portion of the auditory nerve proximal to the brainstem; wave III: cochlear nucleus; wave IV: superior olivary complex; wave V: lateral lemniscus; wave VI: inferior colliculus; wave VII: medial geniculate body.^4^

BAER testing is used routinely in medical practice, given its reproducibility and locating properties. The main aim is to support routine audiological procedures, aiding in the diagnosis of auditory problems that are difficult to assess reliably.

A variety of acoustic stimuli may be used to obtain electrical responses from the brainstem. Clicks are the most frequently used stimuli; clicks may be produced in a wide frequency range, which makes it possible to stimulate more fibers. Frequency selectivity, however, is not possible; the working range is higher frequencies (3,000 to 6,000 Hz).1 Acoustic stimuli such as tone bursts and tone pips may be used in order to obtain frequency-specific responses.^3^

Tone bursts make it possible to obtain relatively narrow frequency range responses, particularly at lower frequencies.^5^

A few authors^6^ affirm that the use of tone bursts in BAER testing provides precision and clinical usefulness when estimating auditory sensitivity at 500 to 4,000 Hz in children and adults. Electrophysiological thresholds obtained with tone bursts are similar to pure tone thresholds, although higher at 500 Hz than at 4,000 Hz.

On the other hand, various papers7-10 have reported the poor quality of BAER responses based on tone bursts, particularly at 500 Hz, where waves are complex, difficult to see and with significantly variable responses. These studies^7^, ^9^ contain few subjects that have electrophysiological thresholds below 70 dBHL, suggesting that the clinical use of tone bursts is questionable.

The response quality at 1,000 Hz obtained from tone bursts appears to be somewhat superior to the response quality at 500 Hz. It is still necessary, however, to use a 30 to 40 dBHL correction factor for the resulting thresholds, 10 suggesting that tone bursts appear to be inadequate for routine clinical use, considering the compatibility between pure tone and electrophysiological thresholds.

Various published papers have used clicks in BAER testing. There are, however, few papers on the standardization of tone burst responses.

The aims of this study were to verify the occurrence of waves I, III and V, and their latency times for a tone burst acoustic stimulus at 500, 1,000, 2,000 and 4,000 Hz at 80 dB NA in audiologically normal subjects aged between 18 and 40 years. Another aim was to compare the electrophysiological thresholds obtained from tone burst stimulation with those obtained in audiologic and psychoacoustic assessments.

## SERIES AND METHODS

The Research Ethics Committee (CAPPesq) of the clinical board of the Sao Paulo Medical School Clinical Hospital (FMUSP) approved this study on 27/01/05, under the research protocol number 1089/04.

Audiological, electrophysiological and psychoacoustic evaluations were done of 40 adult subjects, 20 male and 20 female, aged between 18 and 40 years.

Participants signed a free informed consent form that described the procedures. Clinical history taking was followed by the audiological evaluation, composed by the procedures below: inspection of the external auditory canal with a Heine otoscope, tone and voice audiometry, and acoustic immittance measurement with Grason-Stadler GSI 61 and GSI 68 audiometers and a GSI 33 immittance meter. Normal auditory thresholds were those values between 0 and 25 dBHL at 250 to 8,000 Hz.

The electrophysiological assessment was done with BAER testing using tone bursts at 500, 1,000, 2,000 and 4,000 Hz. A Biologic Traveler Express device, calibrated according to ANSI S3.7-1996 norms, was used.

Abrasive paste was used to clean the skin and electrodes were placed using an electrolytic paste and adhesive tape on the vertex and right and left mastoids.

Electrode impedance values, which were to be below 5 Kohms, were checked. The acoustic stimulus was presented through TDH 39 supra-aural headphones.

The initial tone burst stimulus was 80 dBHL to measure waves I, III and V and their latency times. The intensity was reduced in 20 dB steps until wave V was no longer visible, to investigate the electrophysiological thresholds. We then increased the intensity in 10 dB steps until reaching the lowest intensity in which wave V became visible at its lowest amplitude; this was considered the electrophysiological threshold. There were 1,500 stimuli presented per stimulation. Stimulation was done twice for each intensity to check the reproducibility of tracings and the presence of a response.^3^

Investigation of the tone burst psychoacoustic threshold was done during the electrophysiological testing procedure. This was done to check whether subjects were hearing the acoustic stimulus even in the absence of a BAER; the psychoacoustic threshold was defined as the lowest intensity at which subjects heard tone bursts at 500, 1,000, 2,000 and 4,000 Hz.

## RESULTS

The ANOVA test and the equality of two proportions test were used to analyze the data. The confidence interval technique for the mean and the proportion was used to complement the descriptive analysis.

A significance level of 0.05 (5%) was used and intervals were constructed based on a 95% statistical confidence level.

Waves I and III at 80 dBHL were not observed at 500, 1,000, 2,000 and 4,000 Hz in both sexes.

There were no proportional differences between ears in both sexes in the percentage of occurrence of wave V at 80 dBHL at 500, 1,000, 2,000 and 4,000 Hz. The occurrence of wave V, therefore, was done for both sexes at all of the frequencies that were investigated. A response was considered as absent for a specific frequency when no response was seen in at least one ear (Table 1). There was no proportional difference between sexes for the occurrence of wave V at any frequency that was tested.

**Table 1:** Distribution of the occurrence of wave V at 80 dBHL in males and Femaleales at 500, 1,000, 2,000 and 4,000 Hz.

Wave V	500 Hz		1000 Hz		2000 Hz		4000 Hz	
	Male	Female	Male	Female	Male	Female	Male	Female
Qty	36	36	40	40	40	40	39	40
%	90,0%	90,0%	100,0%	100,0%	100,0%	100,0%	97,5%	100,0%
var	9,3%	9,3%	0,0%	0,0%	0,0%	0,0%	4,8%	0,0%
p-value	1,000		- x -		- x -		0,314	

Key Var: variation rate

There was no statistically significant difference between right and left ear wave V latency in both sexes at the frequencies that were investigated. Table 2 shows the wave V latencies at 80 dBHL for males and females at the frequencies that were investigated.

**Table 2:** Comparison of wave V latencies at 80 dBHL in males and Femaleales.

Wave V latency	500 Hz		1000 Hz		2000 Hz		4000 Hz	
	Male	Female	Male	Female	Male	Female	Male	Female
Minimum	7,18	7,18	7,25	7,02	6,86	6,71	6,79	6,08
Maximum	8,97	9,75	9,36	9,28	8,27	9,28	8,5	7,49
Size	36	36	40	40	40	40	39	40
Lower limit	7,89	7,65	7,75	7,46	7,39	7,13	7,20	6,88
Upper limit	8,20	8,02	8,03	7,76	7,59	7,40	7,43	7,04
Mean	8,05	7,84	7,89	7,61	7,49	7,26	7,31	6,96
Median	8,11	7,92	7,76	7,41	7,41	7,18	7,25	6,94
Standard deviation	0,48	0,57	0,45	0,49	0,33	0,44	0,36	0,26
p-value	0,097		0,010*		0,012*		<0,001*	

**Key:** Fem: Female, *: statistically significant p-value.

There were statistically significant mean differences in wave V latency between males and females at 1,000, 2,000 and 4,000 Hz (p-vales 0.010; 0.012; <0.001). The mean wave V latency was higher in males compared to females at all of these frequencies.

There was no statistically significant difference in the audibility, electrophysiological and psychoacoustic thresholds between right and left ears in males and females at the frequencies that were tested.

We were thus able to group together data from both ears to yield final mean audibility, electrophysiological and psychoacoustic thresholds. A comparison of audibility, electrophysiological and psychoacoustic thresholds in males and females at 500, 1,000, 2,000 and 4,000 Hz is presented below (Tables 3, 4, 5 and 6).

**Table 3:** Comparison of the audibility, electrophysiological and psychoacoustic thresholds in males and Femaleales at 500 Hz.

500 Hz	AT		ET		PT	
	Male	Female	Male	Female	Male	Female
Minimum	0	0	40	40	15	5
Maximum	15	20	80	80	40	45
Size	40	40	34	36	40	40
Lower limit	4,77	3,59	60,41	50,31	27,76	23,40
Upper limit	7,98	6,16	67,24	58,02	31,99	28,85
Mean	6,38	4,88	63,82	54,17	29,88	26,13
Median	5	5	60	50	30	25
Standard deviation	5,19	4,16	10,15	11,80	6,84	8,81
p-value	0,158		<0,001*		0,037*	

**Key:** Male: Male, Fem: Female, AT: Audibility threshold, ET: Electrophysiological threshold, PT: Psychoacoustic threshold, *: statistically significant p-value.

**Table 4:** Comparison of the audibility, electrophysiological and psychoacoustic thresholds in males and females at 1,000 Hz.

1000 Hz	AT		ET		PT	
	Male	Female	Male	Female	Male	Female
Minimum	0	0	50	30	5	5
Maximum	15	15	80	80	35	40
Size	40	40	39	40	40	40
Lower limit	3,01	3,44	62,52	50,89	19,40	18,60
Upper limit	5,74	5,81	68,76	57,61	23,85	23,15
Mean	4,38	4,63	65,64	54,25	21,63	20,88
Median	5	5	60	50	25	20
Standard deviation	4,41	3,82	9,95	10,83	7,20	7,33
p-value	0,787		<0,001*		0,645	

**Key:** Male: Male, Fem: Female, AT: Audibility threshold, ET: Electrophysiological threshold, PT: Psychoacoustic threshold, *: statistically significant p-value.

**Table 5:** Comparison of the audibility, electrophysiological and psychoacoustic thresholds in males and Femaleales at 2,000 Hz.

2000 Hz	AT		ET		PT	
	Male	Female	Male	Female	Male	Female
Minimum	0	0	40	30	0	0
Maximum	20	10	70	70	30	30
Size	40	40	40	40	40	40
Lower limit	2,68	2,74	56,02	46,53	10,21	10,87
Upper limit	6,12	5,01	61,48	52,47	14,54	15,13
Mean	4,40	3,88	58,75	49,50	12,38	13,00
Median	3	5	60	50	10	10
Standard deviation	5,55	3,67	8,83	9,59	6,98	6,87
p-value	0,619		<0,001*		0,688	

**Key:** Male: Male, Fem: Female, AT: Audibility threshold, ET: Electrophysiological threshold, PT: Psychoacoustic threshold, *statistically significant p-value.

**Table 6:** Comparison of the audibility, electrophysiological and psychoacoustic thresholds in males and females at 4,000 Hz.

4000 Hz	AT		ET		PT	
	Male	Female	Male	Female	Male	Female
Minimum	0	0	40	20	0	0
Maximum	25	20	80	70	25	20
Size	40	40	39	40	40	40
Lower limit	4,13	2,00	54,92	44,54	4,66	3,80
Upper limit	8,12	4,75	61,49	50,46	8,09	7,45
Mean	6,13	3,38	58,21	47,50	6,38	5,63
Median	5	2,5	60	50	5	5
Standard deviation	6,45	4,44	10,48	9,54	5,55	5,90
p-value	0,029*		<0,001*		0,560	

**Key:** Male: Male, Fem: Female, AT: Audibility threshold, ET: Electrophysiological threshold, PT: Psychoacoustic threshold, *: statistically significant p-value.

Mean electrophysiological and psychoacoustic thresholds were higher in males compared to females at 500 Hz (p-value <0.001 and 0.037). Males had the highest mean electrophysiological thresholds at 1000 and 2000 Hz (p-value <0.001 at both frequencies). Mean electrophysiological and auditory thresholds in males were higher compared to females at 4,000 Hz (p-value <0.001 and 0.029).

A last analysis compared electrophysiological, psychoacoustic and audibility thresholds in males and females at each frequency that was tested (Tables 7, 8, 9 and 10).

**Table 7:** Comparação entre os limiares de audibilidade, eETtrofisiológico e psicoacústico em 500 Hz.

500Hz	Male			Female		
	AT	ET	PT	AT	ET	PT
Minimum	0	40	15	0	40	5
Maximum	15	80	40	20	80	45
Size	40	34	40	40	36	40
Lower limit	4,77	60,41	27,76	3,59	50,31	23,40
Upper limit	7,98	67,24	31,99	6,16	58,02	28,85
Mean	6,38	63,82	29,88	4,88	54,17	26,13
Median	5	60	30	5	50	25
Standard deviation	5,19	10,15	6,84	4,16	11,80	8,81
p-value	<0,001*			<0,001*		

500 Hz	AT	ET	
Male	ET	<0,001*	
	PT	<0,001*	<0,001*
Female	ET	<0,001*	
	PT	<0,001*	<0,001*

**Key:** AT: Audibility threshold, ET: Electrophysiological threshold, PT: Psychoacoustic threshold, *: statistically significant p-value.

**Table 8:** Comparison of the audibility, electrophysiological and psychoacoustic thresholds at 1,000 Hz.

1000 Hz	Male			Female		
	AT	ET	PT	AT	ET	PT
Minimum	0	50	5	0	30	5
Maximum	15	80	35	15	80	40
Size	40	39	40	40	40	40
Lower limit	3,01	62,52	19,40	3,44	50,89	18,60
Upper limit	5,74	68,76	23,85	5,81	57,61	23,15
Mean	4,38	65,64	21,63	4,63	54,25	20,88
Median	5	60	25	5	50	20
Standard deviation	4,41	9,95	7,20	3,82	10,83	7,33
p-value	<0,001*			<0,001*		

1000 Hz	AT	ET	
Male	ET	<0,001*	
	PT	<0,001*	<0,001*
Female	ET	<0,001*	
	PT	<0,001*	<0,001*

**Key:** AT: Audibility threshold, ET: Electrophysiological threshold, PT: Psychoacoustic threshold, *: statistically significant p-value.

**Table 9:** Comparison of the audibility, electrophysiological and psychoacoustic thresholds at 2,000 Hz.

2000 Hz	Male			Female		
	AT	ET	PT	AT	ET	PT
Minimum	0	40	0	0	30	0
Maximum	20	70	30	10	70	30
Size	40	40	40	40	40	40
Lower limit	2,68	56,02	10,21	2,74	46,53	10,87
Upper limit	6,12	61,48	14,54	5,01	52,47	15,13
Mean	4,40	58,75	12,38	3,88	49,50	13,00
Median	3	60	10	5	50	10
Standard deviation	5,55	8,83	6,98	3,67	9,59	6,87
p-value	<0,001*			<0,001*		

2000 Hz	AT	ET	
Male	ET	<0,001*	
	PT	<0,001*	<0,001*
Female	ET	<0,001*	
	PT	<0,001*	<0,001*

**Key:** AT: Audibility threshold, ET: Electrophysiological threshold, PT: Psychoacoustic threshold, *: statistically significant p-value.

**Table 10:** Comparison of the audibility, electrophysiological and psychoacoustic thresholds at 4,000 Hz.

4000 Hz	Male			Female		
	AT	ET	PT	AT	ET	PT
Minimum	0	40	0	0	20	0
Maximum	25	80	25	20	70	20
Size	40	39	40	40	40	40
Lower limit	4,13	54,92	4,66	2,00	44,54	3,80
Upper limit	8,12	61,49	8,09	4,75	50,46	7,45
Mean	6,13	58,21	6,38	3,38	47,50	5,63
Median	5	60	5	2,5	50	5
Standard deviation	6,45	10,48	5,55	4,44	9,54	5,90
p-value	<0,001*			<0,001*		

4000 Hz	AT	ET	
Male	ET	<0,001*	
	PT	0,989	<0,001*
Female	ET	<0,001*	
	PT	0,322	<0,001*

**Key:** AT: Audibility threshold, ET: Electrophysiological threshold, PT: Psychoacoustic threshold, *: statistically significant p-value.

The audibility, electrophysiological and psychoacoustic thresholds were statistically different from each other in males and females at 500, 1,000 and 2,000 Hz (p-value <0.001 in all three frequencies). The electrophysiological threshold was statistically different from the audibility and psychoacoustic threshold at 4,000 Hz (p-value <0.001), however the last two thresholds were statistically equal to each other in males and females.

## DISCUSSION

In this study waves I and III were not seen at 80 dBHL, as in a paper by Ribeiro (2002),11 who also did not find waves I and III and interpeaks I-III, III-V and I-V in term and preterm newborns, even at high intensities.

There were no statistically significant differences between right and left ears in any of the analyses.

Table 1 shows the occurrence of wave V at 80 dBHL; no statistically significant difference was seen between sexes.

In the current study the occurrence rate of wave V at 1,000 and 2,000 Hz was 100% in both sexes. At 500 Hz the response rate was 90% in both sexes; the response rate at 4,000 Hz was 97.5% in males and 100% in females. Davis e Hirsh (1976),^7^ Laukli et al. (1988),^9^ Conijn et al. (1993),12 Ribeiro (2002),11 Stueve and O´Rourke (2003)13 and Moreira et al. (2005)^14^ have reported that wave V using tone burst stimulation is difficult to visualize even at high intensities. In this study, however, wave V was harder to identify at 500 Hz than at other frequencies; Sininger and Abdala (1996)^15^ justify this possibility by lower neural synchronism at lower frequencies.

There is a significant statistical difference in wave V latency at 80 dBHL between sexes at frequencies over 1,000 Hz with lower mean values in females, as shown on Table 2. Munhoz et al. (2003)^16^ have stated that wave I, III and V latencies in BAER testing using clicks tend to occur earlier in females; according to these authors, wave V latency is measured on average 0.2ms earlier in females. More rapid cochlear responses in females may explain these findings, which would affect the brainstem response (Don et al., 1994).^17^

The wave V latency value decreased as the frequency increased in both sexes. Neely et al. (1988),^18^ and Nagao and Matas (2001)19 reached similar results and stated that low frequencies - that are located on the apex of the cochlea - cover more distance compared to high frequencies, which results in a delay of auditory evoked potential waves at low frequencies. This would explain a higher wave V latency at low frequencies compared to middle and high frequencies. Ribeiro11 also found higher wave V latency values at lower frequencies in newborn subjects.

In the current study electrophysiological thresholds decrease as the frequency increases in both sexes (from 1,000 to 2,000 Hz and from 2,000 to 4,000 Hz); these results are similar to those of Beattie et al. (1996)^20^ and Cone-Wesson et al. (2002),^6^ who also found decreased electrophysiological thresholds at higher frequencies. According to Sininger and Abdala (1996),^15^ increased electrophysiological threshold values at lower frequencies may be explained by lower neural synchronism and the difficulty of visualizing tracings and at these frequencies.

Our data show that males had statistically significant higher electrophysiological thresholds compared to females at all frequencies. Sininger and Abdala (1998)^21^ found similar results in audiologically normal newborn and adult subjects, also demonstrating improved electrophysiological thresholds in females. The authors explained these findings as due to a shorter cochlea in females and more rapid auditory deterioration in males.

Electrophysiological, audibility and psychoacoustic thresholds were compared with each other in all of the four frequencies that were investigated (Tables 7, 8, 9 and 10). Statistically significant differences were found at 500, 1,000 and 2,000 Hz in all of the three thresholds in both sexes. Audibility and psychoacoustic thresholds did not differ at 4,000 Hz, although both were different from the electrophysiological threshold.

Mean electrophysiological and psychoacoustic threshold differences in males and females increased as the frequency was raised. In the current study this may be explained by the finding that psychoacoustic thresholds decrease as frequencies increase, which may be compared to electrophysiological threshold decreases.

The difference between mean electrophysiological and audibility thresholds decreases as frequencies increase in both male and females. Audibility thresholds had constant values at all frequencies, while electrophysiological thresholds decreased. No published paper was found that included the comparisons between the three thresholds made in the current study. We therefore compared these results partially according to findings in other papers.

Our findings are similar to those of Davis and Hirsh (1976),^7^ Laukli et al. (1988),^9^ Fjermedal and Laukli (1989),^10^ and Sininger and Abdala (1996),^15^ who reported a considerable difference between audibility thresholds and electrophysiological thresholds in normal adults and children, particularly below 1,000 Hz; this difference was lowest at 2,000 Hz.9 In these papers,^7^, ^9^, ^10^, ^15^ this difference was about 30 to 40 dBSL; our findings showed a higher difference of about 40 to 50 dBSL in females and 50 to 60 dBSL in males. Sininger and Abdala (1996)^15^ explain the incompatible audibility and electrophysiological threshold results as being due to lower neural synchronism in the below-1,000 Hz frequency region. According to these authors, this problem may be partially solved by adequate equipment calibration.

In our study we also found differences between psychoacoustic and audibility thresholds, where psychoacoustic thresholds were higher. This may be due to the fact that the BAER testing room was not completely acoustically isolated and environmental noise may have altered psychoacoustic thresholds.

Not only was the acoustic room not completely isolated from external noise, but also most of the subjects were awake during testing; there may have been electrical interference due to movement during the test, given that testing lasted over one hour. These issues may have interfered on electrophysiological and psychoacoustic thresholds.

Conijn et al. (1993),^12^ Conijn et al. (1990),22 Stapells (2000),^23^ and Beattie and Rochverger (2001)^24^ pointed to a few difficulties in BAER testing using tone bursts. These issues include the type of high-pass filter that is used, the ambient electrical and acoustic treatment, equipment calibration, the time taken to record electrophysiological thresholds and the state of drowsiness of patients during testing. As a result of these conditions, electrophysiological threshold tracings may be of poorer quality and tend to be found at higher levels than those for audibility thresholds.

The technical issues and the time taken to record electrophysiological thresholds using tone bursts reduce the clinical applicability of this test in adults. Ribeiro (2002),^11^ Stapells (2000),^23^ and Stapells et al. (1995)^2^5 did not reach this conclusion, as in their papers testing was done on children; in these papers, audibility and electrophysiological thresholds were in agreement, probably due to more favorable testing conditions.

## CONCLUSION

The following conclusions were reached based on the data in this paper:

1- Waves I and III at 80 dBHL at 500, 1,000, 2,000 and 4,000 Hz were not seen in males and females.
2- The occurrence of wave V in males and females at 1,000 Hz and 2,000 Hz was 100%. The response rate was 90% in males and females at 500 Hz; the response rate was 97.5% in males and 100% in females at 4,000 Hz.
3- Wave V latency and the electrophysiological threshold decreased as the frequency increased in males and females.
4- Electrophysiological thresholds were higher in males compared to females at all frequencies. Psychoacoustic thresholds were higher in males compared to females at 500 Hz.
5- Auditory, electrophysiological and psychoacoustic threshold were statistically different in males and females at 500, 1,000 and 2,000 Hz. Auditory and psychoacoustic thresholds did not differ from each other at 4,000 Hz, although both were different from the electrophysiological threshold.
6- Although BAER testing using tone bursts is applicable to the clinical setting, our results show a significant difference between electrophysiological and audibility thresholds in an adult population. Further studies are needed to standardize testing techniques and the results of BAER testing using tone bursts.
